# Practical Guidelines for Approaching the Implementation of Neural Networks on FPGA for PAPR Reduction in Vehicular Networks

**DOI:** 10.3390/s19010116

**Published:** 2018-12-31

**Authors:** Abdelhamid Louliej, Younes Jabrane, Víctor P. Gil Jiménez, Ana García Armada

**Affiliations:** 1 GECOS Lab, National School of Applied Sciences, Cadi Ayyad University, 40000 Marrakech, Morocco; a.louliej@uca.ma (A.L.); y.jabrane@uca.ma (Y.J.); 2 Department of Signal Theory and Communications, University Carlos III of Madrid, Leganés, 28911 Madrid, Spain; agarcia@tsc.uc3m.es

**Keywords:** ECMA-368, peak to average power ratio, neural networks, FPGA implementation

## Abstract

Nowadays, the sensor community has become wireless, increasing their potential and applications. In particular, these emerging technologies are promising for vehicles’ communications (V2V) to dramatically reduce the number of fatal roadway accidents by providing early warnings. The ECMA-368 wireless communication standard has been developed and used in wireless sensor networks and it is also proposed to be used in vehicular networks. It adopts Multiband Orthogonal Frequency Division Multiplexing (MB-OFDM) technology to transmit data. However, the large power envelope fluctuation of OFDM signals limits the power efficiency of the High Power Amplifier (HPA) due to nonlinear distortion. This is especially important for mobile broadband wireless and sensors in vehicular networks. Many algorithms have been proposed for solving this drawback. However, complexity and implementations are usually an issue in real developments. In this paper, the implementation of a novel architecture based on multilayer perceptron artificial neural networks on a Field Programmable Gate Array (FPGA) chip is evaluated and some guidelines are drawn suitable for vehicular communications. The proposed implementation improves performance in terms of Peak to Average Power Ratio (PAPR) reduction, distortion and Bit Error Rate (BER) with much lower complexity. Two different chips have been used, namely, Xilinx and Altera and a comparison is also provided. As a conclusion, the proposed implementation allows a minimal consumption of the resources jointly with a higher maximum frequency, higher performance and lower complexity.

## 1. Introduction

Recently, ultra wideband (UWB) has been used for radar or sensing in vehicular communications that play an essential role into operational areas in Smart Cities [1,2]—in addition, in military communications and niche applications for its number of advantages that make it attractive for consumer communications applications (low cost, resistant to severe multipath and good time resolution) [3]. In vehicular communications [4], those advantages are especially relevant since transmissions must be reliable for safety applications. Moreover, the requirements are very strict in terms of power consumption and data rate due to the critical applications. In February 2002, the Federal Communications Commission (FCC) has implemented a regulation authorizing the use of UWB technology for telecommunications consumer in the United States by assigning a frequency band of 7.5 GHz not subject to licensing (FCC 02-48), the FCC opened the door for a very high data rate (beyond Gbps). The terminology UWB refers at first to waveforms without carriers (carrier-free) made of very short pulses [5]. In this context, a commonly accepted definition is that these signals have a fractional bandwidth (FB), greater than 0.25 with a frequency bandwidth greater than 500 MHz [6]. The calculation of fractional bandwidth is indicated in Equation (1)

(1) $$F_{B}=\frac{\left(F_{H}-F_{L}\right)}{F_{C}}\;\;with\;\;F_{C}=\frac{\left(F_{H}+F_{L}\right)}{2},$$

where $F_{H}$ is the upper frequency, $F_{L}$ is the Lower Frequency and $F_{C}$ is the Center Frequency.

Orthogonal Frequency Division Multiplexing (OFDM) technology is a modulation technique adopted in many broadcast standards. This is due to many advantages of OFDM: Robustness to frequency fading (very important in V2V - Vehicle to Vehicle communications) [7], resilient to intersymbol interference (ISI), spectrum efficiency and simple channel equalization. The ECMA(European Computer Manufacturers Association)–368 Standard also specifies a Multiband Orthogonal Frequency Division Modulation (MB-OFDM) scheme to transmit information for a wireless personal area network (WPAN). Despite the advantages of OFDM, however, it is characterized by large power envelope fluctuations, thereby a loss of power efficiency is obtained when signals go through the High Power Amplifier (HPA) due to the nonlinearity. This is particularly important in wireless sensor networks where the energy constraints are very strict. In the literature, there are many proposals to reduce or mitigate this problem in OFDM signals such as [8,9,10]. Active Constellation Extension (ACE) is one of the best choices to solve this drawback that is able to obtain a signal with an arbitrarily low Peak to Average Power Ratio (PAPR) with the adequate number of iterations. The ACE method modifies and expands the constellation points within an allowable region without affecting the demodulation slicer, and thus it does not need side information. In [10], different algorithms to achieve PAPR reduction through ACE are provided. The main problem with these algorithms is the complexity and convergence mainly due to high number of iterations, although arbitrarily low PAPR signals can be obtained. In [11], a neural network (NN) technique, referred to as Multilayer Perceptrons (MLPs), to obtain signals with low envelope fluctuations has been developed. Indeed, NN have been widely applied in solving optimization problems [12,13,14]. In the case of the PAPR proposal in [11], the NN were trained with the Approximate Gradient Projection (AGP) from ACE [10] and thus the result is an NN that generates from the original signal another one with similar characteristics as ACE but without its complexity and in one shot. The algorithm in [11] reduces the complexity, but, from the point of view of the implementation in a real system, only theoretical results are given. Although some ideas are devised, in order to be useful in real implementations, several key aspects need to be analyzed such as bandwidth, maximum data rate and physical space consumption. For this reason, in this paper, all these issues are analyzed and optimized. In addition, some guidelines for a generic Software Defined Radio (SDR) implementation of algorithms are also outlined. There are many papers where a description of implementation of a specific algorithm is shown; however, to the best knowledge of the authors, no other papers address from this perspective the real implementation of the PAPR algorithm [15].

Since, during the last several decades, the digital signal processing capabilities have been dramatically increased with the Digital Signal Processor (DSP) and the Field Programmable Gate Array (FPGA), the novel devices are able to run complex algorithms and thus many improvements can be obtained. The adoption of these circuits promises an easy adjustment of bandwidth, gain and rate, giving rise to more flexible radio systems. Thus, algorithms that were too complex for being implemented can be afforded now with the consequent improvement on the system performance. FPGAs with their intrinsically parallel structures become the preferred technology choice to overcome the processing and flexibility requirements for future generation systems. The logical outcome of these trends is, without a doubt, digital signal processing carried out by software, known as SDR [16,17,18]. In addition, SDR architectures allow a wide range of design techniques to achieve fully flexible transmission/reception systems for future applications. This is especially interesting in vehicular communications because the community is still researching the best transmission scheme and standard. Moreover, it will depend on the application and, since in a vehicular network there are many applications such as passive safety, active safety, entertainment, information, or optimization among others, SDR is a very promising approach. In order to pave the way to this SDR paradigm, powerful hardware is becoming popular for mobile communications devices and thus novel algorithms can be used, such as the one proposed in this paper. However, even with the new powerful advanced architectures and hardware designs, there are limitations on complexity, size, operating frequency, bandwidth and delay that need to be taken into account. Thus, the implementation should be optimized to obtain a useful system architecture.

In this paper, the novel system structure and implementation of advanced algorithms for PAPR reduction proposed in [11] is described and analyzed, and some conclusions and guidelines for similar designs are drawn from the optimization process.

The paper is organized as follows: Section 2 presents the ECMA-368 standard which is also advised for vehicular systems. The proposed solution is carried out in Section 3. In Section 4, an implementation of the proposed solution is described and analyzed. Then, results are presented and discussed in Section 5. Finally, some conclusions are drawn in Section 6. Notation: in this paper, the following notation is used. Lower faced and capital letters denote time-domain and frequency-domain, respectively. The sub index indicates if the signal is real part or imaginary part because NN can only operate with real-valued numbers and super index is used to specify the algorithm or model being used.

## 2. ECMA-368 Standard

**Figure 1:**
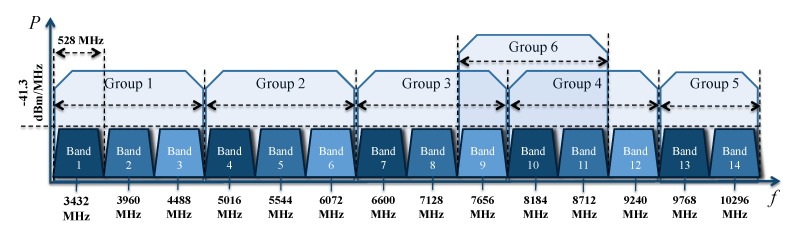
The ECMA-368 band groups.

The physical layer of ultra wideband using MB-OFDM is described by ECMA-368 in Wireless personal area (WPAN) and is also advised for vehicular networks. It is allocated into the unlicensed 3.1–10.6 GHz frequency band. It also adopts 53,3 Mb/s, 80 Mb/s, 106,7 Mb/s, 160 Mb/s, 200 Mb/s, 320 Mb/s, 400 Mb/s, and 480 Mb/s as data rates. In Figure 1, the ECMA-368 band is shown; this band is split into six groups of bands. Band groups 1 to 4 contain three bands each, covering the bands 1 to 12. Band group 5 consists two bands 13 and 14. Band group 6 contains the bands 9, 10 and 11. Band group 1 is used for mandatory mode and the rest of the bands groups are dedicated for future use. The center frequency $f_{c}$ is related to the band number $n_{b}$ by: $f_{c}=2904+528*n_{b},\;n_{b}=1\ldots 14$ (MHz) [19]. The transmitted MB-OFDM symbols are time-interleaved across the 14 bands according to the specified time-frequency code (TFC) [19].

Table 1 presents the MB-OFDM characteristics. An IFFT (Inverse fast Fourier transform) of 128 points generates the MB-OFDM symbol. Between 128 sub-carriers, 100 are for data, 12 pilots, 10 guard subcarriers, five zero guards and the DC. The subcarrier frequency spacing Δf = 4.125 MHz can fulfill the requirement of orthogonality in the OFDM system. The data rates are tuned by four possible forward error correction (FEC) codings, which are convolutional codes using 1/3, 1/2, 5/8 and 3/4 as coding rates.

**Figure 2:**
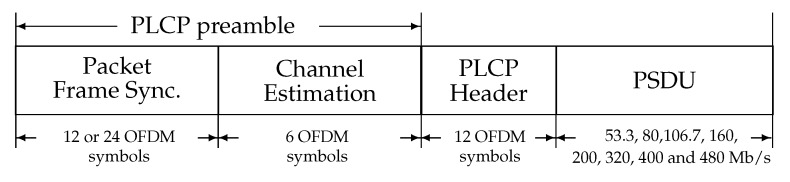
PHY frame structure.

Eventually, the duration of each transmitted MB-OFDM symbol containing 165 samples is Ts = 312.5 ns. Figure 2 [19] shows how the PHY (Physical) service interface and the MAC are connected by using a Physical Layer Convergence Protocol (PLCP) sublayer, and how a PSDU (PHY Service Data Unit) is converted to a PPDU (PLCP Packet Data Unit).

The PLCP Preamble: contains the packet of synchronization and channel estimation sequences. The PLCP Header: contains information needed on both PHY and MAC layers, for example: MAC header, PHY header, Reed–Solomon parity bits. The PSDU: contains essentially the data packets.

ECMA-368 uses two types of modulations:

1. QPSK modulation used with data rates of 53.3 up to 200 Mb/s.
2. Dual-Carrier Modulation (DCM) used with data rates of 320 up to 480 Mb/s, in this modulation the Bits are divided into groups of 200 bits, and further grouped into 50 groups of 4 reordered bits. Then, the DCM modulation uses a matrix H to execute a mapping of the two QPSK symbols into two DCM symbols which form two 16-QAM constellations [20].

## 3. The Algorithm

Once the ECM-368 standard and the PAPR problem has been briefly described, in this section, a solution is devised. In the literature, there are many proposal for PAPR reduction in OFDM-based signals, as explained at the introduction. Among them, the ACE algorithm is one of the best options to obtain a signal with arbitrarily low PAPR with the adequate number of iterations (usually high). The ACE method modifies and expands the constellation points within an allowable region without affecting the demodulation slicer, and thus, it does not need side information.

**Figure 3:**
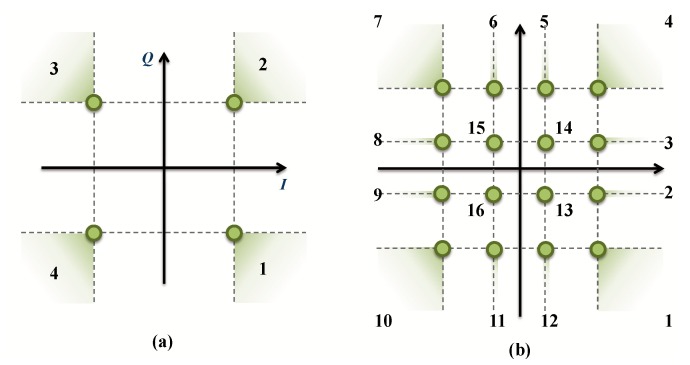
QPSK and DCM ACE processing.

As it is described in [10], the ACE-AGP is an iterative algorithm. In the following, the algorithm will be summarized. Every constellation point is moved within the allowable region away from its initial position in an iterative procedure. As example, for QPSK and 16-QAM cases, in Figure 3, the allowable regions are depicted (shadowed). We first clip the signal peaks in the time-domain signal and observe what happens in the frequency-domain. If points moved into an allowable region, the algorithm keeps them, if not, they are restored to their previous positions and the time-domain signal is evaluated again. Mathematically, it can be summarized as follows:

1. Use IFFT to obtain *x* from the modulated signal *X*. Reset the number of iterations *j* to 0.
2. Clip all $|x^{j}\left[k\right]|\geq B$ (where B represents the signal’s magnitude), then x[k] becomes:
  (2) $$\bar{x}\left[k\right]=\left\{\begin{array}{cc} x^{j}\left[k\right] & \;|x^{j}\left[k\right]|\leq B,\\ Be^{i\theta [k]} & \;|x^{j}\left[k\right]|>B. \end{array}\right.$$
3. Calculate the added clipped signal portion:
  (3) $$c_{clip}\left[k\right]=\bar{x}\left[k\right]-x^{j}\left[k\right].$$
4. Obtain $C_{clip}$ by applying an FFT on $c_{clip}$
5. The only $C_{clip}$ components with acceptable extension directions respecting the given sub-channel constellations are kept, the rest is set to 0.
  (4) $$C_{clip}\left[k\right]=\left\{\begin{array}{cc} 0 & \;(|ℜ\left(C_{clip}\left[k\right]\right)|+|ℜ\left(x^{j}\left[k\right]\right)|)\leq Q,\\ 0 & \;(|ℑ\left(C_{clip}\left[k\right]\right)|+|ℑ\left(x^{j}\left[k\right]\right)|)\leq Q, \end{array}\right.$$
  where *Q* represents the allowable regions of QPSK modulation.
6. Obtain $c_{clipnew}$ using IFFT and compute:
  (5) $$X_{new}^{j}\left[k\right]=X^{j}\left[k\right]+c_{clipnew}^{j}\left[k\right].$$
7. If the target PAPR requirement is not achieved or the maximum number of iterations (*j*) is not reached, go to Step 2. Otherwise, the algorithm finishes and the output is the obtained signal.

For less complexity and fast convergence, authors in [11] proposed a novel NN architecture designed and trained to obtain low PAPR signals by synthesizing the behavior of the ACE-AGP algorithm, but with much less complexity. The idea is to design an NN that would be able to obtain similar signals than ACE-AGP but with less complexity and without the iterative process that takes time and resources. To do this, as explained in [11], the NN is trained with time-domain and frequency-domain signals obtained from ACE-AGP as references. Thus, once the NN is trained, it is able to generate similar signals (with low PAPR) directly from the original ones without the iterative process and with less complexity. Thus, authors in [11] developed NN models based on the time-domain and frequency-domain OFDM signal, respectively, and provide the theoretical framework. Here, a brief description is provided for clarity purposes.

The time-domain complex base-band OFDM signal can be expressed as:

(6) $$x\left[n\right]=\frac{1}{\sqrt{N}}\sum_{k=0}^{N}S_{k}\exp\left(\frac{2\pi nk}{N}\right),$$

where $S_{k}$ is the complex modulated symbol at kth sub-carrier (usually M-QAM), and *N* is the number of sub-carriers. In order to obtain a modified low PAPR version of x[n], we use an NN.

The feed-forward network is one of the most used classes between several ANN architectures. It has one or more hidden layers using nonlinear functions and an output layer with linear functions. These ANNs are known as Multi Layer Perceptrons (MLPs) trained with different algorithms where the Levenberg–Marquardt one has been used to optimize the Backpropagation training technique so as to get fast and good convergence.

**Figure 4:**
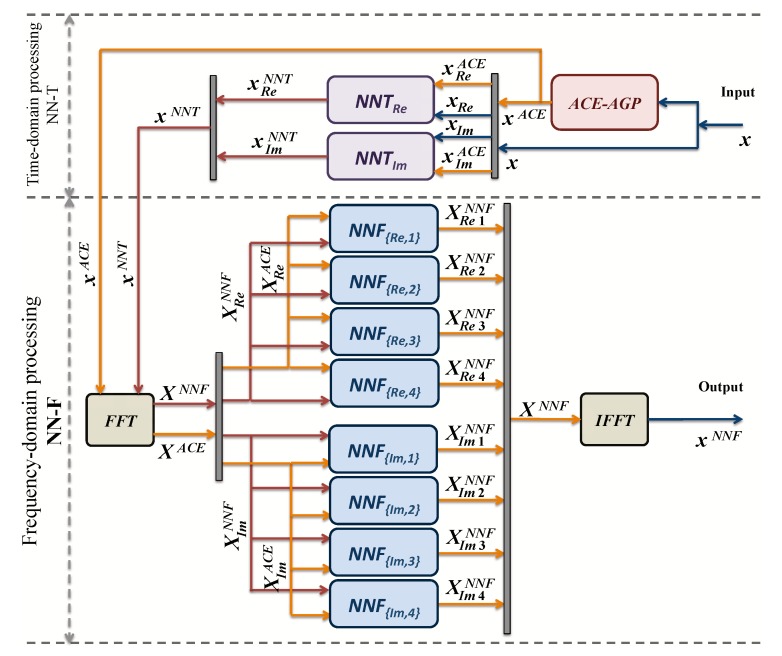
Time-domain and frequency-domain Neural Network training.

The idea is that the NN learns how to obtain low PAPR signals from original OFDM symbols. Thus, the NN is trained showing as input the original OFDM symbol and as output the desired low PAPR signal obtained with ACE-AGP algorithm. However, as explained in [11], this trainee must be carried out in the time and frequency domain at the same time because there is relevant information in both domains. From the time-domain signal, the NN learns about how the low PAPR signals look but from the frequency-domain, the NN acquires the knowledge of the allowable regions where constellation points can be moved. Thus, we need to train the NN architecture, simultaneously in both domains. This procedure is described as follows [11]:

1. Use the original time-domain data *x* as input to the ACE-AGP algorithm to obtain $x^{ACE}$, i.e., a signal with reduced envelope fluctuations.
2. Decompose the original data x into real and imaginary parts ($x_{Re}$,$x_{Im}$), and the ACE-AGP output $x^{ACE}$ into ($x_{Re}^{ACE}$,$x_{Im}^{ACE}$).
3. Train the time-domain models NNT by using signals $\left[x_{Re},x_{Re}^{ACE}\right]$ and $\left[x_{Im},x_{Im}^{ACE}\right]$ to obtain the real and imaginary NN models: $NNT_{Re}$ and $NNT_{Im}$.
4. Obtain $x_{Re}^{NNT}$ and $x_{Im}^{NNT}$ by using the former NN models with input signals $x_{Re}$ and $x_{Im}$.
5. Apply FFT on $x_{Re}^{NNT}$ and $x_{Im}^{NNT}$ to obtain the frequency-domain signal $X^{NNF}$.
6. Split the training samples $X^{NNF}$ in the four constellation regions in order to train eight NNs. We will divide the signal in two sets: 1st set concerning real parts and 2nd set concerning the imaginary parts, as it can be seen in Figure 4.
7. Train the first set of NNs by $X_{Re}^{NNF}$ to generate the NN models in time-domain $NNF_{RE,1}$, $NNF_{RE,2}$, $NNF_{RE,3}$ and $NNF_{RE,4}$ for each quadrant.
8. Train the second set of NNs by $X_{Im}^{NNF}$ to generate the NN models in frequency-domain $NNF_{Im,1}$, $NNF_{Im,2}$, $NNF_{Im,3}$ and $NNF_{Im,4}$ for each quadrant.

This training procedure is depicted in Figure 4. It should be highlighted that, once the NNs are trained offline, the ACE algorithm is not used anymore [11].

Once the NN is trained offline, the procedure for obtaining the low PAPR signals from the original OFDM symbol in one shot is the following:

1. Decompose the original time-domain signal *x* into real and imaginary parts ($x_{Re}$,$x_{Im}$).
2. Feed $x_{Re}$ and $x_{Im}$ into the already off-line trained neural networks $NNT_{Re}$ and $NNT_{Im}$, respectively, to obtain $x_{Re}^{NNT}$ and $x_{Im}^{NNT}$.
3. Apply FFT on $x_{Re}^{NNT}$ and $x_{Im}^{NNT}$ to obtain the frequency-domain signal $X^{NNF}$.
4. Separate the obtained $X^{NNF}$ in the four constellation regions.
5. Feed with these signals to the eight frequency-domain Neural networks $NNF_{RE,1}$, $NNF_{RE,2}$, $NNF_{RE,3}$, $NNF_{RE,4}$ and $NNF_{IM,1}$, $NNF_{IM,2}$, $NNF_{IM,3}$, and $NNF_{IM,4}$.
6. Perform an IFFT to obtain the output low PAPR signal $x^{NNF}$

As it can be observed, the ACE-AGP algorithm is no longer needed and the signal is produced without any iteration, i.e., no delay, which is critical in many vehicular transmissions, especially in safety applications.

### 3.1. New Architecture

Before the implementation, further simplifications should be done in order to reduce the complexity, increase bandwidth, but, at the same time, without affecting results and performance. Taking into account the symmetry of the problem, as it is shown in Figure 3a, the number of NNs can be reduced to only two frequency-domain models in the QPSK cases, i.e., $NNF_{RE,1}$, $NNF_{Im,2}$.

**Figure 5:**
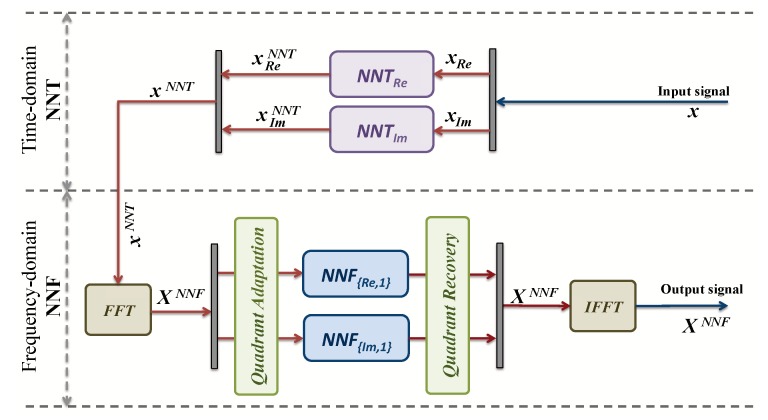
New time-domain and frequency-domain NN for QPSK.

**Figure 6:**
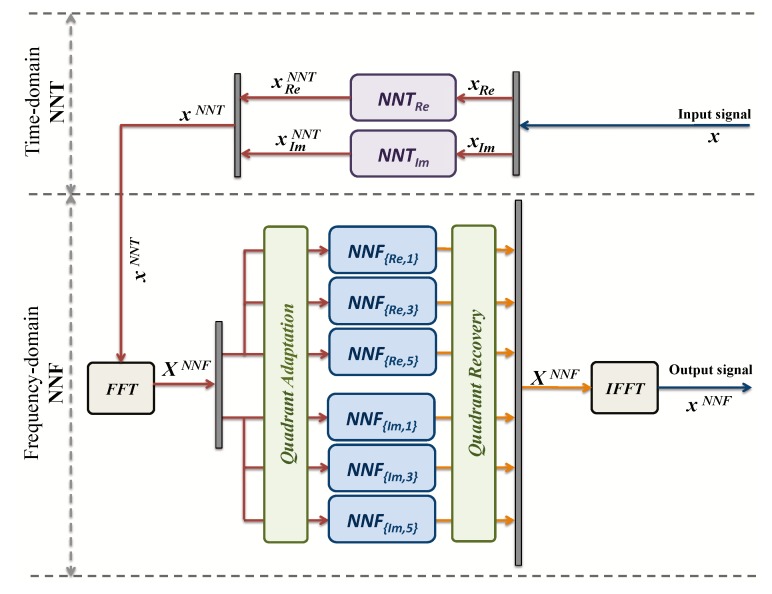
New time-domain and frequency-domain NN for DCM.

For this purpose, new blocks “Quadrant Adaptation” and “Quadrant Recovery” are needed, at the transmitter and the receiver, respectively, for constellation quadrants adaptation to/from the operating frequency-domain models. The architecture is shown in Figure 5. This new architecture will save space and energy. In addition, in DCM cases, for the same reason (Figure 3b), the number of neural networks can be reduced from 24 to 6. In fact, two models $NNF_{RE,1}$, $NNF_{Im,2}$ can be used for regions 1, 4, 7 and 10. Two other models $NNF_{RE,3}$, $NNF_{Im,3}$ for regions 2, 3, 8 and 9. In addition, in regions 5, 6, 11 and 12, two models $NNF_{RE,5}$, $NNF_{Im,5}$ are also used. Finally, regions 13, 14, 15 and 16 do not undergo any processes since interior points cannot be moved [10]. Figure 6 shows the new architecture for DCM modulation.

Each NN constituting the architectures proposed in Figure 5 and Figure 6 is in three layers:

1. An input layer: acquires the input signal of the system.
2. A hidden layer: contains two neurons adopting triangular function activation.
3. An output layer: contains a single neuron with a linear activation function.

**Figure 7:**
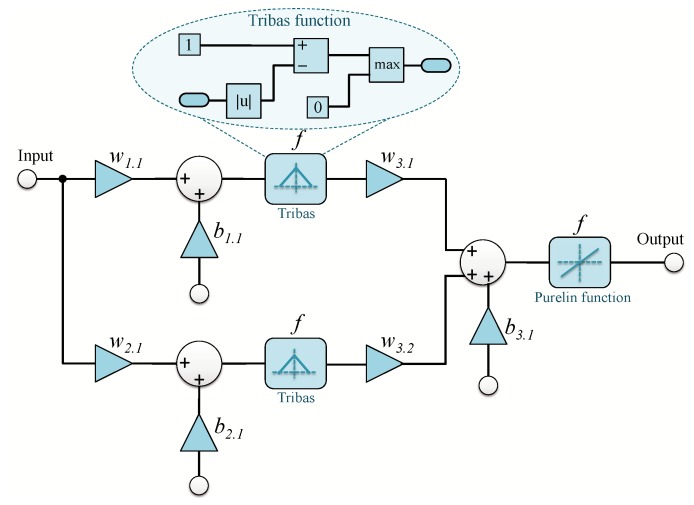
NN model design.

The designed NN is shown in Figure 7.

### 3.2. Complexity Analysis

From Figure 7, we conclude that, for N subcarriers, the time-domain NN models’ complexity in both QPSK and DCM, in terms of number of integer multiplications and integer additions is 14 × N and 12 × N, respectively. The frequency-domain NN models complexity, in terms of number of integer multiplications and integer additions is 14 × 4 × N and 12 × 4 × N for QPSK, and 14 × 12 × N and 12 × 12 × N for DCM. In the proposed frequency-domain NN models, the complexity is relative to the type of modulation used, so the number of integer multiplications and integer additions is 14 × N and 12 × N for QPSK, and 14 × 3 × N and 12 × 3 × N for DCM, respectively (Table 2).

## 4. Implementation of the Proposed Solution

There are in the market several platforms for implementing embedded systems [21]. In our case, two different platforms have been used and compared, namely Nutaq (SFF-SDR) and Altera (Stratix II EP2S180). The two platforms integrate FPGAs of Xilinx and Altera, respectively. We used an FPGA instead of a DSP for the benefits it offers. Indeed, an FPGA allows a higher frequency, supporting higher bit rates and providing real-time processing.

**Figure 8:**
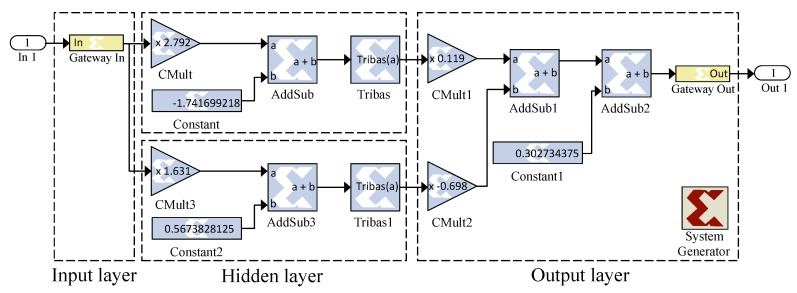
Implemented NN using a Xilinx system generator.

**Figure 9:**
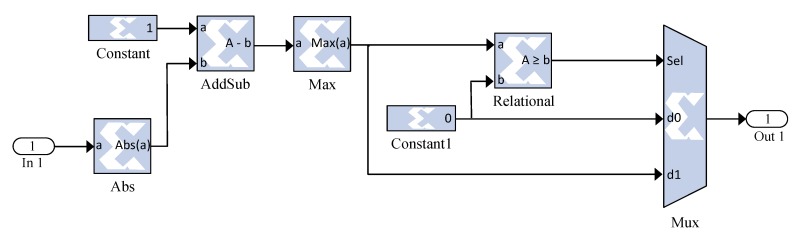
Implemented triangular function using a Xilinx system generator.

The training process of time and frequency-domain NN is done in an offline way; therefore, only their layers will be implemented on an FPGA circuit (without the learning algorithm). Figure 8 and Figure 9 illustrate the architecture of a NN as well as the activation function implemented during our development, respectively.

It is worth noting that the real and imaginary parts of the signal are separately processed; thus, this architecture will be duplicated in the case of the time-domain solution and multiplied by the number of constellation areas treated in the case of the frequency-domain solution.

**Figure 10:**
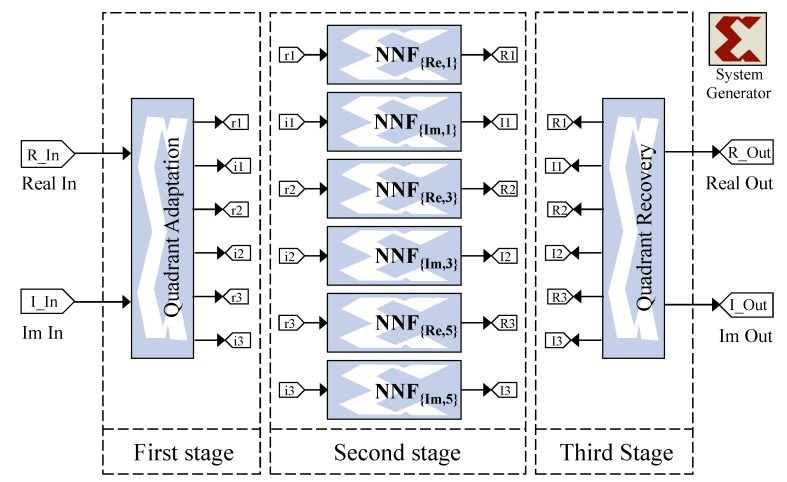
Implemented frequency-domain NN using a Xilinx system generator.

We first test the implementation of our proposed solution on OFDM signals with different numbers of subcarriers for QPSK and 16-QAM modulations. In order to represent each OFDM sample, we adopted signed fixed-point representation that provides a compromise between the traditional and the floating-point representations. Indeed, it allows higher computational speeds and minimal resource consumption. Following a statistical study carried out on the proposed NN regarding the minimum and maximum values of signals, we found that each sample can be represented with 16 bits: a sign bit, five bits for the integer part and finally 10 for the fractional part. In contrast to the time-domain NN, the frequency-domain NN does not allow the reduction of the power fluctuations present in an OFDM signal; on the other hand, it retains the triangular shape of the modulation constellation imposed by the ACE-AGP algorithm. Recall that, in the case of QPSK modulation, the number of frequency-domain NN is two, whereas, for a 16-QAM modulation, this number is six. Figure 10 illustrates the implementation of the frequency-domain NN for 16-QAM modulation.

The implemented architecture in the case of 16-QAM is subdivided into three different stages as shown in Figure 10. The first stage allows for determining the belonging of a point to a quadrant of the constellation and adapting it to frequency-domain NN. The second stage consists of three blocks, each one grouping two NNs allowing a different treatment of the real and imaginary parts to ensure a proper expansion. The last stage allows for recovering the original position of the constellation point. In case of QPSK modulation, the same stages will be used, with the difference that the second one will contain one block instead of three.

**Figure 11:**
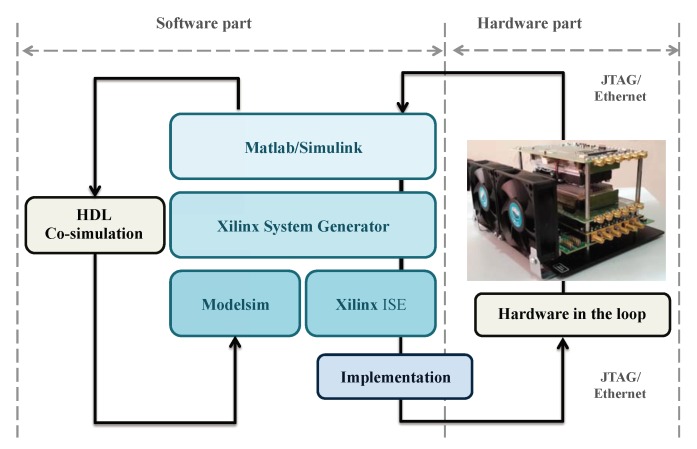
Hardware co-simulation.

For comparing the achieved performance with that obtained by simulation, we choose a JTAG (joint test action group) hardware co-simulation [22]. This feature allows for simulating the whole or part of a design implemented directly on an FPGA platform. This approach also makes it possible to accelerate the simulation of a complex design and to verify its correct functioning in the hardware. The reason behind the use of a hardware co-simulation is to minimize the development time while avoiding implementing the entire OFDM transmission and reception system on an FPGA platform. In fact, only the proposed NN will be implemented on a hardware platform while the rest will be emulated by software. At each clock cycle, the software sends a data frame to the hardware for processing. The communication between the software and the hardware is carried out either by a JTAG or Ethernet cable for more speed (Figure 11).

## 5. Results and Discussion

In order to evaluate our implementation, a set of performance criteria has been adopted, namely, the gain in cubic metric reduction, the Bit Error Rate (BER) degradation and the resources’ consumption.

In a communication system, the BER is a critical parameter; thus, some experiments have been conducted to evaluate it. For this purpose, the physical layer of the ECMA-368 standard [19] has been used. It describes the physical layer of an Ultra Wideband (UWB) communication system intended for Wireless Personal Area Network (WPAN), using a band of frequencies not subject to a license between 3.1 GHz and 10.6 GHz. It supports different bit rates: 53.3 Mbps, 80 Mbps, 106.7 Mbps, 160 Mbps, 200 Mbps, 320 Mbps, 400 Mbps and 480 Mbps. This standard adopts Multiband Orthogonal Frequency Division Multiplexing (MB-OFDM) technology [19].

In addition to OFDM, it has a frequency hopping provided by a Time-Frequency Coding (TFC). Each ECMA-368 symbol consists of 128 subcarriers, which span a bandwidth of 528 MHz.

### 5.1. Cubic Metric

The conventional metric used to measure power fluctuations in an OFDM signal is Peak-to-Average Power Ratio (PAPR). However, the latter does not take into account the distortion induced by HPA. For this reason, the Third Generation Partnership Project (3GPP) proposed the cubic metric [23,24]. It is mathematically defined as follows:

(7) $$CM=\frac{RCM-RCM_{ref}}{K},$$

where RCM is the raw cubic metric, which is defined for a signal *x* as follows:

(8) $$RCM=20log_{10}\left(\sqrt{E\left\{{\left(\frac{x(t)}{\sqrt{x(t)}}\right)}^{3}\right\}}\right)\;\left(\mathrm{dB}\right).$$

$RCM_{ref}$ is the RCM reference that for OFDM takes the value 1.52 dB and *K* is 1.56 [24].

#### 5.1.1. OFDM Signals’ Case

**Figure 12:**
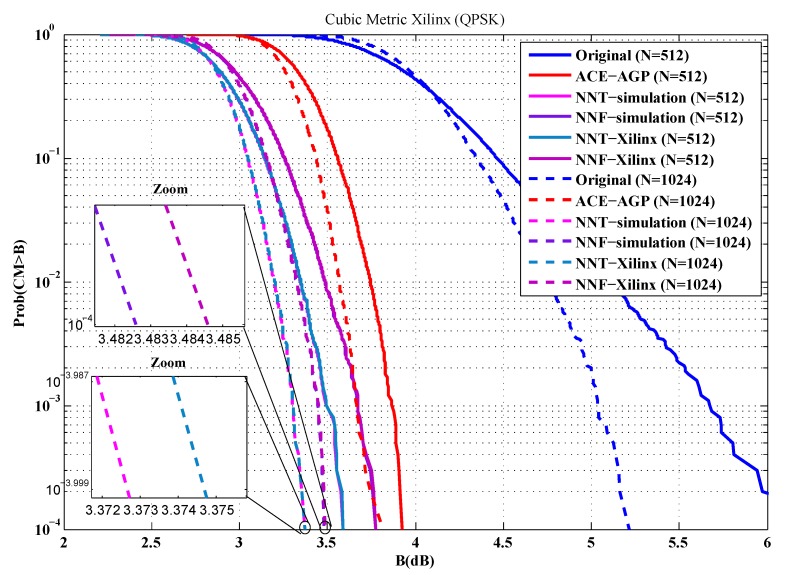
Cubic metric reduction in the case of QPSK modulation (Xilinx FPGA).

**Figure 13:**
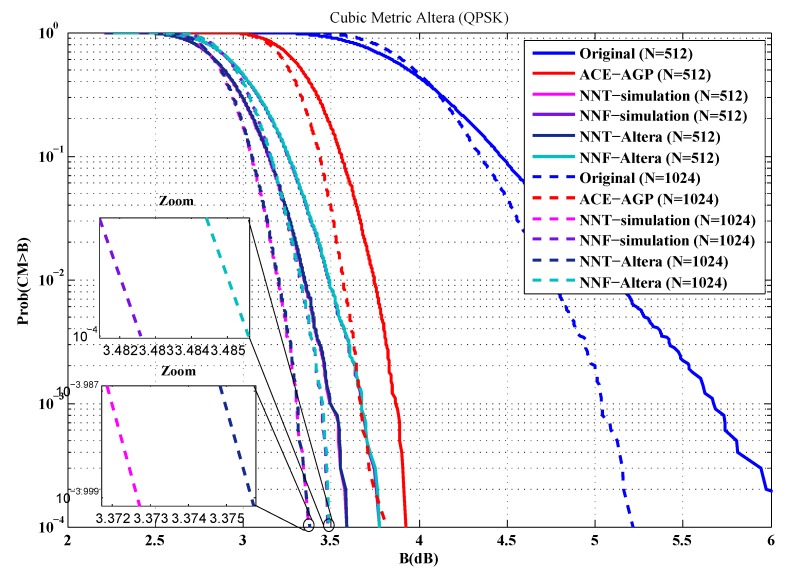
Cubic metric reduction in the case of QPSK modulation (Altera FPGA).

**Figure 14:**
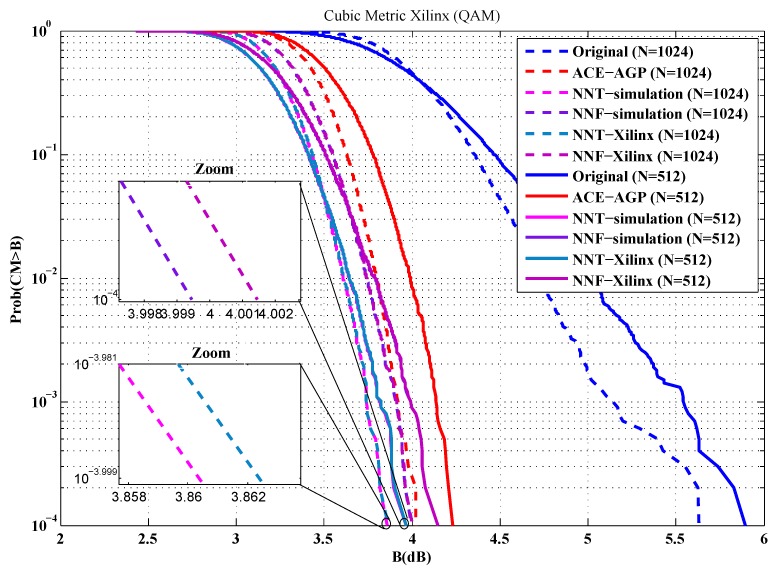
Cubic metric reduction in the case of 16-QAM modulation (Xilinx FPGA).

**Figure 15:**
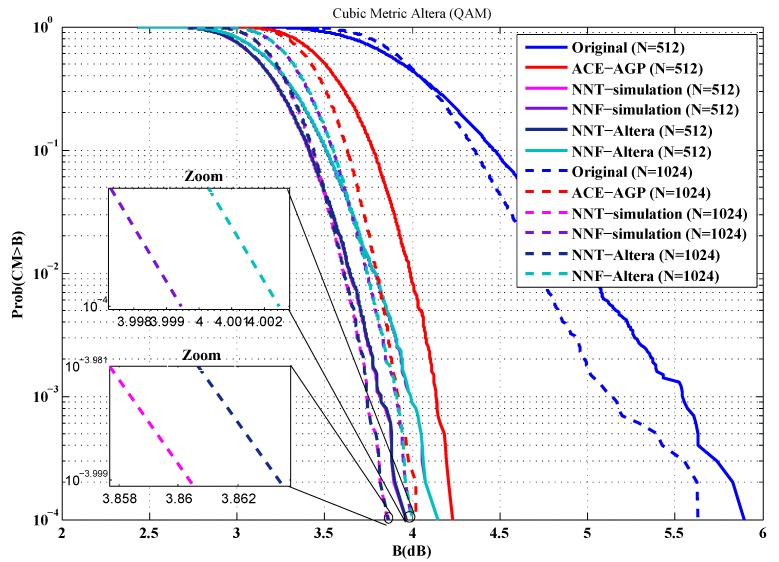
Cubic metric reduction in the case of 16-QAM modulation (Altera FPGA).

To evaluate the performance of our implementations, the first metric used is the cubic metric. For this purpose, a series of measurements of the cubic metric over 10,000 OFDM symbols are carried out. The QPSK and 16-QAM modulated OFDM symbols are generated randomly for *n* = 512 and 1024 sub-carriers. Figure 12, Figure 13, Figure 14 and Figure 15 show the obtained results.

From these figures, it is clear that the results provided by implementing the proposed solutions are faithful to those obtained by simulation. Indeed, in the case of an implementation on Xilinx FPGA chip, the average error in reduction of the cubic metric is 0.002 dB, while, for an implementation on the Altera FPGA chip, it is equal to 0.003 dB. The small errors observed can be justified by the truncation errors caused by the fixed-point representation.

#### 5.1.2. ECMA-368 Signals Case

**Figure 16:**
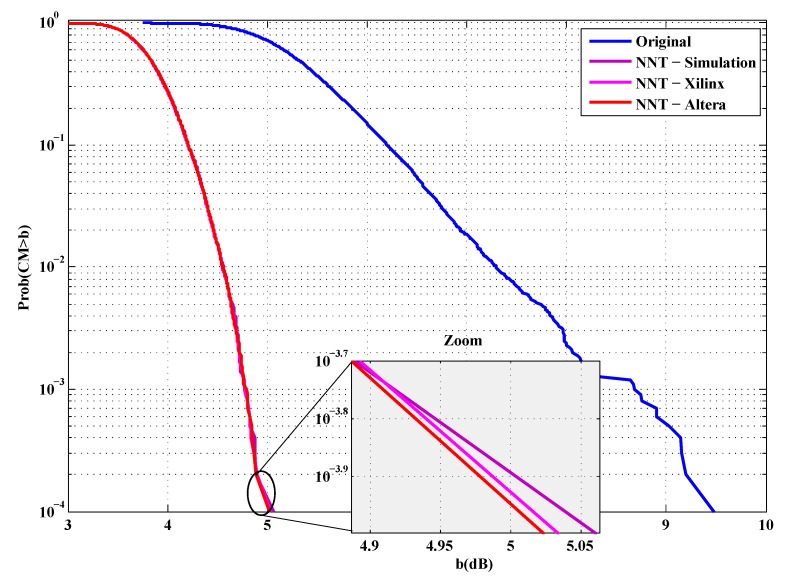
ECMA-368 cubic metric reduction in the case of QPSK modulation.

**Figure 17:**
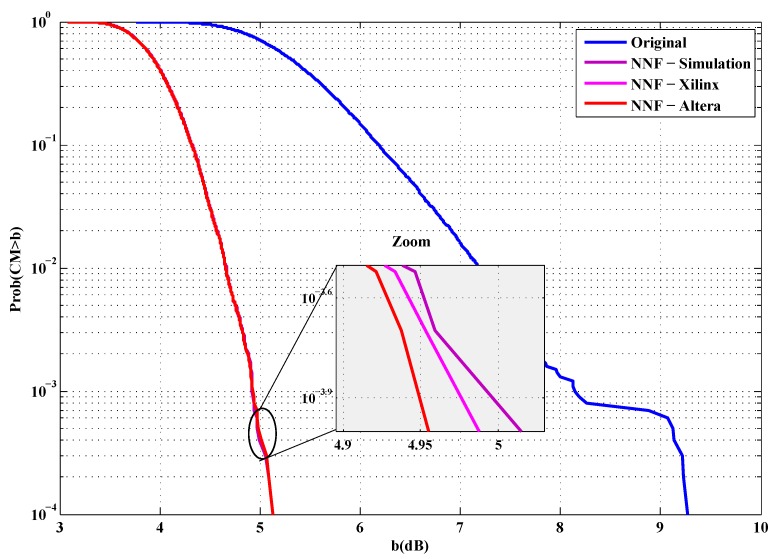
ECMA-368 cubic metric reduction in the case of DCM modulation.

Before drawing the BER, the cubic metric of the ECMA-368 standard is plotted. First, in Figure 16 and Figure 17, the cubic metric is plotted to verify that the implementation is working properly.

It is clear that our implementation allows a good reduction of cubic metric of ECMA-368 signals.

### 5.2. Bit Error Rate

To plot the ECMA-368 BER curves, we used the UWB multipath channel based on the Saleh and Valenzula model proposed by IEEE 802.15.3a [25,26,27,28]. In this channel, the multipath components, denoted as rays (paths), arrive at the receiver in groups of clusters. A double Poisson process can represent this phenomenon. The IEEE 802.15.3.a considers four Channel Models (CM1 to CM4) used in this paper and configured as shown in Table 3.

**Figure 18:**
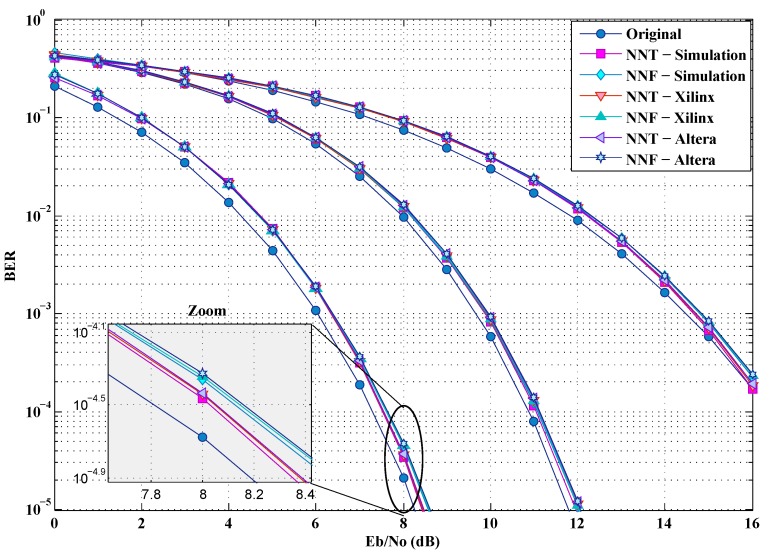
BER of the implementations for a CM1 channel.

**Figure 19:**
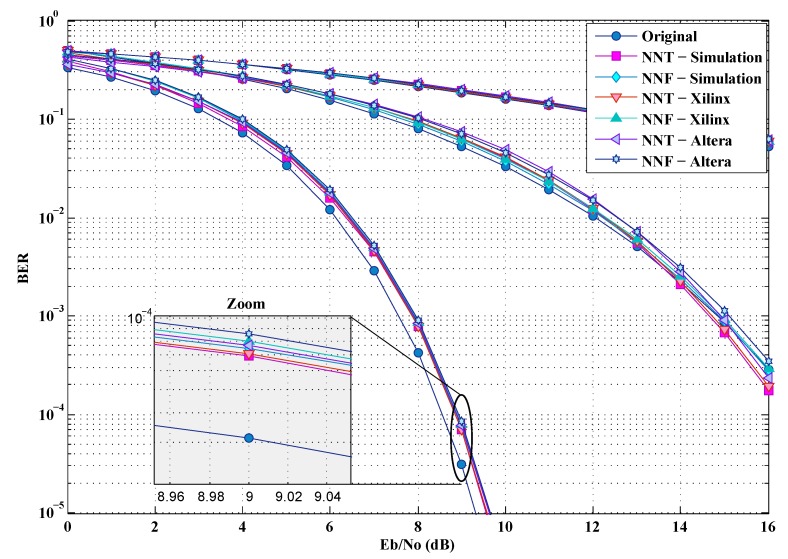
BER of the implementations for a CM4 channel.

To analyze the BER by simulation, we use the UWB channel model and Additive white Gaussian noise (AWGN) is added at the Rx. These simulations adopt three different data rates 53.3, 200 and 480 Mbps, and the Time Frequency Codes TFC1 of the band group number 1 [19]. This will allow us to test the proposed solutions according to the modulations imposed by the standard (QPSK and DCM) taking into account the full range of possible rates. It should be noted that, besides the real implementation on FPGA (Xillinx or Altera), some simulations have also been carried out in order to check and validate the implementations. Figure 18 and Figure 19 show, in comparison with the simulations, that the hardware implementations have very little impact on the BER. In fact, this slight degradation of the BER can be justified by the truncation error caused by a fixed-point calculation.

### 5.3. Resources’ Consumption

**Figure 20:**
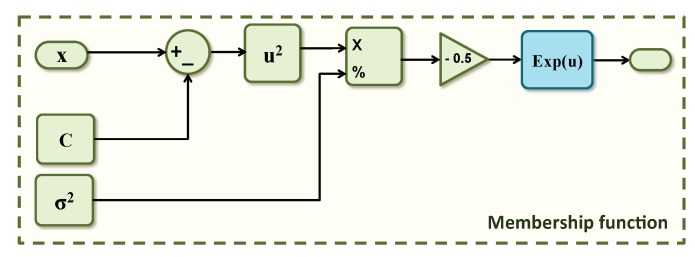
Gaussian membership function.

To reduce PAPR in OFDM transmitted signal, in [29], the authors proposed another alternative solution based on Adaptive Neuro-Fuzzy Inference System (ANFIS). To map inputs to the membership functions, the proposed ANFIS [29] uses a Gaussian membership function based on an exponential function as shown in Figure 20, where *C* and σ are, respectively, the center and the variance of the Gaussian membership function.

To implement the exponential function, in [30], the authors proposed a new approximation method based on Taylor series. Altera provides also in their intellectual property core (IP core) library a floating-point exponential function (ALTFP_EXP) [31]. Table 4 provides a comparison of consumed resources between the triangular function presented in Figure 7, ALTFP_EXP and the proposed approximation in [30].

From Table 4, we can easily notice that the implemented triangular function is faster and consumes less FPGA resources (for example less adaptive lookup tables (ALUT)) than the implemented exponential functions. For these reasons, in this article, we opted for neural networks’ architecture with triangular activation function instead of ANFIS architecture or any other architectures based on exponential functions.

Among the hardware solutions, we quoted the GC1115 proposed by Texas Instruments (Dallas, TX, USA) [32]. It operates on a maximum of 32 MHz bandwidth and allows the reduction of the Crest Factor (CF) in the Wideband Code Division Multiple Access (WCDMA) and OFDM signals. The drawback of this type of solution lies in the fact that it requires a hardware implementation and therefore the total modification of the electronic circuit. To overcome this disadvantage, the two competitors Xilinx and Altera offer software defined solutions that can be implemented on FPGA chips. In fact, Xilinx integrates in its Intellectual Property Core (IP Core) library a kernel named Peak Cancellation Crest Factor Reduction (PC-CFR) [33], which reduces the crest factor of the following communication standards: CDMA2000, WCDMA, WiMAX and LTE, while Altera offers in its library the Crest Factor Reduction (CFR) module [34] destined to the same standards. Unfortunately, these two solutions are subject to very costly licenses. As long as all these solutions allow processing in the time domain, a comparison with the time-domain NN is covered out. This comparison will allow us to estimate the resources exploited on different FPGA chips as well as the maximum frequency supported by each solution. Table 5 and Table 6 show the results of this comparison.

From these tables, we can conclude that our solution based on time-domain NN is much less resource-consuming than former solutions and implementations. In the case of an implementation on Xilinx chips, we note that the number of DSP blocks is zero. This is justified by the fact that all the multiplication operations are realized by logical elements. With regard to the use of the Look-Up Tables (LUT), the time-domain NN allows for 60%, 53.7% and 55% reductions, respectively, for Virtex-5, Spartan-6 and Virtex-6. Since all weighting coefficients and biases are stored directly on the FPGA logic circuits, our solution does not use any memory blocks, which impacts the resources consumption and the operating frequency directly. For Virtex-6, the maximum frequency of our solution is 540 MHz, thus far exceeding the 400 MHz provided by the Xilinx solution, which will allow us to support the 528 MHz required by the ECMA-368 standard. In the case of an implementation on Altera chips, we note the use of DSP blocks, so, for a Stratix III, the number of these blocks is estimated to be 16, giving a reduction of 33.3% in favor of our solution while the reduction in the use of LUTs is 82%. From the same tables, it can be seen that, unlike the frequency-domain NN, the time-domain NN is characterized by the same resources’ consumption and the same maximum frequency independently from the type of modulation used.

## 6. Conclusions

In this paper, two new implemented solutions for reducing the high power envelope fluctuations of the OFDM signal in vehicular communications are introduced. The first is in the time-domain to reduce the power fluctuations while the second is carried out in the frequency-domain in order to keep the demodulation slicer intact. To minimize the complexity of the second solution starting from the theoretical design of [11], we reduced the number of NNs by leveraging on the symmetry of the problem. Indeed, in the case of a QPSK modulation, this number has been reduced from 8 to 2, whereas, in the case of a DCM modulation, this number is narrowed from 24 to 6. Some other optimizations have also been developed to reduce size, increase bandwidth and speed up the computations.

The models have been implemented on FPGA circuits and some clues are drawn for future designs. To validate them, we used the cubic metric, the BER and resources’ consumption.

Concerning the cubic metric, a slight error of 0.002 dB is observed in the case of Xilinx and 0.003 dB in the case of Altera with respect to simulations. This is justified by the residual error of the fixed-point calculation adopted by each of these constructors.

To ensure that our implementations do not affect the performance of an OFDM communication system, we have plotted the BER in a real ECMA-368 standard, and, as it has been shown, it fits specifications perfectly.

We compared the proposed solutions with those provided by Xilinx and Altera and we were able to conclude that the time-domain NN allowed a minimum consumption of resources and a higher maximum frequency regardless of the type of modulation. Finally, we have developed and implemented two versions of our algorithms in realistic architectures suitable for vehicular networks, and several guidelines are drawn for future implementations and optimizations in such networks approaching the implementation of OFDM on FPGA for vehicular communications.

## Figures and Tables

**Table 1:** OFDM parameters for the ECMA-368 standard.

Parameters	Value
Number of data subcarriers	100
Number of pilot subcarriers	12
Total of subcarriers used	122
Subcarrier frequency spacing	4.125 MHz
IFFT/FFT period	242.42 ns
Zero padded suffix duration	70.08 ns
Symbol interval	312.5 ns
Number of samples per zero padding suffix	37
Total number of samples per symbol	165
Symbol rate	3.2 MHz
Subcarrier modulation	QPSK or DCM
Code rates	1/3, 1/2, 5/8, 3/4

**Table 2:** Complexity summary for *n* = 128 subcarriers.

Complexity	Temporal NN	Old Frequency NN	Proposed Frequency NN
QPSK			
Integer mults.	1792	7168	1792
Integer adds.	1536	6144	1536
DCM			
Integer mults.	1792	21504	5376
Integer adds.	1536	18432	4608

**Table 3:** UWB channel model parameters.

Model Parameter	CM1	CM2	CM3	CM4	Unit
LOS/NLOS	LOS	NLOS	NLOS	NLOS	-
TX-RX Separation	0–4	0–4	4–10	4–10	m
Cluster rate Λ	0.0233	0.4	0.667	0.0667	1/ns
Ray rate λ	2.5	0.5	2.1	2.1	1/ns
Cluster time decay Γ	7.1	5.5	14	24	ns
Ray time decay Υ	4.3	6.7	7.9	12	ns
$\sigma_{1}$	3.3941	3.3941	3.3941	3.3941	dB
$\sigma_{2}$	3.3941	3.3941	3.3941	3.3941	dB

**Table 4:** Resource consumption of Tribas versus Exponential functions.

Solutions	FPGA Chips	ALUT	DSP Blocks	Memory	Max. Freq. (MHz)
Triangular function	Stratix II	42	0	0	370
Proposed approximation in [30]	Stratix II	154	28	0	280
ALTFP_EXP	Stratix II	1177	35	232	274

**Table 5:** Resource consumption in the case of Xilinx FPGAs.

Solutions	FPGA Chips	RAM Blocks	DSP Blocks	LUTs	Max. Freq. (MHz)
Xilinx PC-CFR V6.0		7	18	2040	335
Time-domain NN (QPSK)	Virtex-5	0	0	887	358
Time-domain NN (DCM)	xc5vlx110-1	0	0	887	358
Frequency-domain NN (QPSK)		0	0	1065	122
Frequency-domain NN (DCM)		0	0	2508	77
Xilinx PC-CFR V6.0		6	18	1690	193
Time-domain NN (QPSK)	Spartan-6	0	0	849	278
Time-domain NN (DCM)	xc6slx100-2	0	0	849	278
Frequency-domain NN (QPSK)		0	0	1039	80
Frequency-domain NN (DCM)		0	0	2386	47
Xilinx PC-CFR V6.0		4	18	1737	400
Time-domain NN (QPSK)	Virtex-6	0	0	849	534
Time-domain NN (DCM)	xc6vlx130t-1	0	0	849	534
Frequency-domain NN (QPSK)		0	0	1039	280
Frequency-domain NN (DCM)		0	0	2386	127

**Table 6:** Resource consumption in the case of Altera FPGAs.

Solutions	FPGA Chips	RAM Blocks	DSP Blocks	LUTs	Max. Freq. (MHz)
Altera CFR		6	12	2801	95
Time-domain NN (QPSK)		0	16	334	311
Time-domain NN (DCM)	Cyclone III	0	16	334	311
Frequency-domain NN (QPSK)		0	24	617	106
Frequency-domain NN (DCM)		0	46	3103	67
Altera CFR		6	20	1922	111
Time-domain NN (QPSK)		0	16	334	311
Time-domain NN (DCM)	Stratix II	0	16	334	311
Frequency-domain NN (QPSK)		0	24	499	106
Frequency-domain NN (DCM)		0	40	1462	67
Altera CFR		6	24	1922	111
Time-domain NN (QPSK)		0	16	334	421
Time-domain NN (DCM)	Stratix III	0	16	334	421
Frequency-domain NN (QPSK)		0	24	499	143
Frequency-domain NN (DCM)		0	60	1462	90
